# Exposure to Electronic Media, Smoking and Alcohol Drinking Among Guyanese Adults

**DOI:** 10.3389/fpubh.2022.862975

**Published:** 2022-06-20

**Authors:** Liming Shao, Ting Zhang, Yanyan Chen, Bishwajit Ghose, Lu Ji

**Affiliations:** ^1^Planning and Development Department, Hangzhou Medical College, Hangzhou, China; ^2^Zhejiang Business College, Hangzhou, China; ^3^Hangzhou Medical College, Hangzhou, China; ^4^Organisation pour l'environnement et Développement Durable, Lomé, Togo; ^5^School of Medicine and Health Management, Huazhong University of Science and Technology, Wuhan, China

**Keywords:** tobacco, alcohol, Guyana, MICs, internet use

## Abstract

Use of electronic media has been shown to be associated with tobacco and alcohol consumption behavior among adult population. Currently, not much is known about the risk factors of tobacco and alcohol consumption in Guyana. The present study aimed to explore the association between exposure to electronic media and tobacco and alcohol consumption by adjusting for the sociodemographic correlates.

**Methods:**

Data were obtained from the sixth round of Guyana Multiple Indicator Cluster Survey (MICS 2019–20). Sample population were 2,208 men and 5,872 women aged between 15 and 49 years. Outcome variables were self-reported lifetime use of tobacco and alcohol, and the main explanatory variables were the use of electronic media such as internet, television and radio. Data were analyzed using descriptive and multivariable regression analysis in Stata version 17.

**Results:**

Overall, 55.5% (95% CI = 53.4, 57.6) men and 44.5% (95% CI = 42.4, 46.6) women reported ever smoking tobacco, while the percentage of ever drinking alcohol was 34.2% (95% CI = 32.9, 35.4) and 65.8% (95% CI = 64.6, 67.1) among men and women, respectively. Internet users had significantly higher odds of ever consuming tobacco and alcohol, however, the association was not uniform across the sociodemographic variables. For smoking, the positive association with internet use was observed for women only (OR = 1.32, 95% CI = 1.09, 1.59), whereas, for alcohol consumption the association with internet use was significantly positive among both men (OR = 1.82, 95% CI = 1.58, 2.09) and women (OR = 1.84, 95% CI = 1.22, 2.70). Men and women who ever tried alcohol drinking had (OR = 4.64, 95% CI = 3.16, 6.82) and (OR = 10.62, 95% CI = 7.83, 14.40) times higher odds of trying tobacco smoking.

**Conclusion:**

Current findings indicate over a quarter of the participants reported ever smoking tobacco and more than three-fifths ever drinking alcohol. Electronic media use, especially that of internet, is a strong predictor of tobacco and alcohol consumption among Guyanese adults. Results also revealed a strong interrelation between alcohol and tobacco smoking, and suggesting that strengthening the tobacco prevention programs may reduce the prevalence of alcohol drinking as well.

## Introduction

Guyana is a middle-income country located in South America with a population of about 0.78 million. During the last 10–15 years, the country has witnessed considerable progress in population health in terms of increased life expectancy, reduction in maternal and child mortality, greater access to healthcare services and availability of improved water and sanitation facilities (1–4). In parallel with these important achievements, the country is also experiencing a rapid rise in the burden of non-communicable diseases (NCDs) such as cardiovascular diseases, stroke, diabetes and cancer (5–7). As such, the country is undergoing a major epidemiological transition from infectious to chronic diseases characterized by changes in lifestyle-related behaviors such as unhealthy dietary behavior and increased consumption and tobacco and alcoholic products. In fact, Guyana has the highest rates of cardiovascular and ischemic heart disease-related deaths compared to other countries in the Americas, and also experienced a 32% rise in the prevalence of hypertension between 2007 and 2017 (5). Studies have shown that both alcohol and tobacco are major risk factors for NCDs (8–11). For instance, tobacco and alcohol use is a risk factor for stroke with a population attributable risk (PAR) of 12.4 and 5.8%, respectively (5, 12). Global Burden of Disease (GBD) study ranks tobacco and alcohol consumption as the leading risk factors for global disease burden (13). According to a World Health Organization report, tobacco and alcohol are the most important modifiable causes of NCD related morbidity and mortality worldwide (14). Identifying the drivers of tobacco and alcohol use at population level is therefore a key step to developing NCD prevention programs.

There is a growing volume of research dedicated to exploring the factors associated with alcohol and tobacco use among adolescent and adult populations across different cultures (15, 16). These factors are generally classified as various demographic (e.g., age, sex), socioeconomic (e.g., education, income) and commercial (e.g., exposure to advertisements) determinants (17–19). Healthcare systems are introducing various policies to address these determinants such as surtaxing tobacco products and communicating public awareness programs (20). One of the most challenging among these determinants is the rising popularity of electronic media that has created the scope for aggressive marketing like never before and targeting the consumers in developing countries who were previous less reachable through traditional marketing strategies. Population-based studies from many countries have shown a significant impact of social media use and consumption of tobacco and alcohol among adults (21–25). Addressing this situation is especially complex because electronic media has also become the main platform for sharing health-related information not only for general population, but also for public health institutions (26, 27). Understanding the association between media use and tobacco and alcohol use is therefore of critical importance among health researchers and practitioners in all countries. To date, no such study has been conducted among the Guyanese population. In this study, we aimed to fill this gap in literature by analyzing cross-sectional data from Guyana Multiple Indicator Cluster Survey (MICS 2019–20) that collected data on smoking and alcohol drinking along with other sociodemographic and media use related variables.

## Methods

### Data Source

Data for this study was obtained from the sixth round of Guyana Multiple Indicator Cluster Survey (MICS 2019–20) which is a part of the global MICS program. The survey was carried out by the Bureau of Statistics with Technical support from the United Nations Children's Fund (UNICEF). The global MICS program was developed by UNICEF in the 1990s as an international household survey program to help countries conduct quality surveys on diverse health and socioeconomic indicators and track progress toward the Millennium Development Goals (MDGs). The surveys employ multistage cluster sampling design to ensure representativeness of the data. The first stage involves selection of enumeration areas across the regions to form primary sampling units (PSUs), and then a systematic selection of households from the PSUs (28). Data are collected using structured questionnaires on eligible men and women aged 15–49 years and children under 5 years of age. The present study included both male (*n* = 2,208) and female (*n* = 5,872) participants.

### Description of Study Variables

The outcome variables were lifetimes use alcohol and tobacco consumption. Participants were asked: “Have you ever tried smoking tobacco?” and “Have you ever tried drinking alcohol.” The answers were coded as “yes” and “no.” The explanatory variables of primary interest were the use of electronic media that are used for health communication such as internet, television and radio. These responses were dichotomized into “internet user” and “non-user.” Frequency of TV watching and listening to radio was categorized as: “Not At All,” “Less Than Once a Week,” “At Least Once a Week,” and “Almost Every Day.” We also included several demographic, socioeconomic factors as potentially confounding variables based on their conceptual correlation with the outcome variables: Age (15–19; 20–24; 25–29; 30–34; 35–39; 40–44; 45–49), Sex (Male; Female), Marital status (Currently Married/In Union; Formerly Married/In Union; Never Married/In Union), Ethnicity (African/Black; Amerindian; East Indian; Mixed Race; Other), Type of residency (Urban; Rural), Education (Primary; Secondary; Higher), Wealth index quintile (Poorest; Second; Middle; Fourth; Richest). MICS surveys do not collect information on individual income, but instead provide a measure household wealth index which is calculated by using principal components analysis that involves assigning scores on individual household possessions, e.g., durable goods, household building material, water, and sanitation to generate factor scores for each item. Households are then ranked based on individual scores to range between poorest, poorer, middle, richer, and richest (29).

### Data Analysis

All analyses were performed using Stata version 17 using the svy command to account for the cluster survey design. We performed cross-tabulations to describe the socio-demographic characteristics of the participants by tobacco and alcohol consumption status along with χ2 bivariate tests showing the statistical significance between the outcome and the explanatory variables. The percentage of tobacco and alcohol consumption across the age groups stratified by the media use variables were presented as bar charts. Following that, we ran a series of regression models to calculate the odds ratios of the associations between tobacco and alcohol consumption with the three media use variables while controlling for the sociodemographic factors. Three separate binary regression models were run for each of the outcome variables (one for the full sample, and two more for male and female samples). The sex-specific analysis was performed to examine whether the associations varied between men and women. For test performance of the six regression models, we reported ROC curve values due to their capacity for diagnostic accuracy (30). The last step of the analysis included three sets of binary regression models to measure the association between tobacco and alcohol consumption. These models were also adjusted for all the sociodemographic and media use variables. A *p*-value of <0.05 was considered statistically significant for all associations.

## Results

Sociodemographic profile of the sample population was presented in Table 1. Overall, 55.5% (95% CI = 53.4, 57.6) men and 44.5% (95% CI = 42.4, 46.6) women reported ever smoking tobacco, while the percentage of ever drinking alcohol was 34.2% (95% CI = 32.9, 35.4) and 65.8% (95% CI = 64.6, 67.1) among men and women, respectively. The pattern of both tobacco and alcohol use differed significantly by age group and sex. A greater percentage of the men and women who reported ever consuming tobacco and alcohol were in the age groups of 25–29 years. The percentages were also higher among those who were currently married, of East Indian origin, residents of rural areas, had secondary level education and from households with lowest wealth quintiles.

**Table 1:** Percentage of sample population reporting ever using tobacco and alcohol.

		**Tobacco use**			**Alcohol use**		
	**Total**	**No**	**Yes**	***P*-value**	**No**	**Yes**	***P*-value**
		72.9 (72.0; 73.9)	27.1 (26.1; 28.0)		29.3 (28.3; 30.3)	70.7 (69.7; 71.7)	
**Ever use internet** (Yes)	57.6 (56.4; 58.8)	56.6 (55.2; 58.0)	60.2 (57.9; 62.6)		46.4 (44.3; 48.6)	62.6 (61.2; 64.0)	
(No)	42.4 (41.2; 43.6)	43.4 (42.0; 44.8)	39.8 (37.4; 42.1)	0.01	53.6 (51.4; 55.7)	37.4 (36.0; 38.8)	0.00
**Frequency of watching TV** (not at all)	23.8 (22.8; 24.7)	24.5 (23.4; 25.6)	21.9 (20.2; 23.6)		29.1 (27.2; 30.9)	21.5 (20.5; 22.6)	
(Less than once a week)	8.7 (8.0; 9.3)	8.7 (8.0; 9.4)	8.5 (7.3; 9.6)		10.1 (8.9; 11.4)	8.1 (7.3; 8.8)	
(At least once a week)	19.2 (18.3; 20.0)	19.6 (18.6; 20.7)	17.9 (16.3; 19.5)		18.1 (16.6; 19.7)	19.6 (18.6; 20.6)	
(Almost every day)	48.4 (47.3; 49.5)	47.2 (45.9; 48.4)	51.8 (49.7; 53.9)	0.00	42.6 (40.7; 44.6)	50.8 (49.5; 52.1)	0.00
**Frequency of listening to radio** (Not At All)	49.1 (48.0; 50.2)	51.2 (49.9; 52.4)	43.5 (41.4; 45.5)		56.4 (54.4; 58.4)	46.0 (44.7; 47.3)	
(Less than once a week)	10.2 (9.5; 10.9)	10.1 (9.3; 10.9)	10.4 (9.1; 11.7)		10.1 (8.9; 11.3)	10.3 (9.5; 11.0)	
(At least once a week)	14.8 (14.0; 15.6)	15.4 (14.5; 16.3)	13.3 (11.9; 14.7)		14.7 (13.2; 16.1)	14.9 (13.9; 15.8)	
(Almost every day)	25.9 (25.0; 26.9)	23.4 (22.3; 24.4)	32.9 (30.9; 34.8)	0.00	18.8 (17.2; 20.4)	28.9 (27.7; 30.0)	0.00
**Age** (15–19)	17.8 (17.0; 18.7)	21.1 (20.0; 22.1)	9.1 (7.9; 10.4)		28.1 (26.3; 29.9)	13.6 (12.7; 14.5)	
(20–24)	18.2 (17.3; 19.0)	18.8 (17.8; 19.8)	16.3 (14.8; 17.9)		16.9 (15.4; 18.5)	18.6 (17.6; 19.6)	
(25–29)	16.5 (15.7; 17.3)	15.9 (14.9; 16.8)	18.3 (16.7; 20.0)		13.1 (11.7; 14.4)	18.0 (17.0; 19.0)	
(30–34)	12.4 (11.7; 13.2)	11.6 (10.8; 12.4)	14.8 (13.3; 16.3)		9.7 (8.5; 10.9)	13.6 (12.7; 14.5)	
(35–39)	11.9 (11.2; 12.6)	11.2 (10.4; 12.0)	14.0 (12.5; 15.4)		10.1 (8.8; 11.3)	12.7 (11.8; 13.5)	
(40–44)	11.8 (11.1; 12.5)	11.2 (10.4; 12.0)	13.3 (11.8; 14.7)		11.1 (9.8; 12.4)	12.0 (11.2; 12.9)	
(45–49)	11.3 (10.6; 12.0)	10.3 (9.5; 11.1)	14.2 (12.7; 15.6)	0.00	11.0 (9.7; 12.2)	11.5 (10.7; 12.3)	0.00
**Sex** (Male)	27.3 (26.4; 28.3)	16.9 (15.9; 17.8)	55.5 (53.4; 57.6)		10.8 (9.6; 12.1)	34.2 (32.9; 35.4)	
(Female)	72.7 (71.7; 73.6)	83.1 (82.2; 84.1)	44.5 (42.4; 46.6)	0.00	89.2 (87.9; 90.4)	65.8 (64.6; 67.1)	0.00
**Marital status** (currently married/in union)	66.2 (65.2; 67.3)	63.6 (62.3; 64.8)	73.5 (71.6; 75.3)		57.9 (55.9; 59.8)	69.7 (68.5; 70.9)	
(Formerly married/in union)	8.5 (7.9; 9.1)	7.9 (7.2; 8.5)	10.1 (8.8; 11.3)		5.9 (5.0; 6.9)	9.5 (8.7; 10.3)	
(Never married/in union)	25.3 (24.3; 26.2)	28.6 (27.4; 29.7)	16.5 (14.9; 18.0)	0.00	36.2 (34.3; 38.1)	20.8 (19.7; 21.8)	0.00
**Ethnicity** (African/black)	24.9 (23.9; 25.8)	25.4 (24.3; 26.5)	23.4 (21.6; 25.2)		20.6 (19.0; 22.3)	26.6 (25.5; 27.8)	
(Amerindian)	20.2 (19.3; 21.1)	20.7 (19.7; 21.8)	18.8 (17.2; 20.4)		27.1 (25.3; 28.9)	17.4 (16.4; 18.4)	
(East Indian)	34.6 (33.5; 35.6)	35.4 (34.2; 36.6)	32.3 (30.3; 34.2)		37.6 (35.7; 39.6)	33.3 (32.1; 34.5)	
(Mixed race)	20.1 (19.2; 20.9)	18.1 (17.1; 19.1)	25.3 (23.5; 27.2)		14.4 (13.0; 15.8)	22.4 (21.4; 23.5)	
(Other)	0.3 (0.2; 0.4)	0.3 (0.2; 0.5)	0.2 (0.0; 0.4)	0.00	0.3 (0.1; 0.5)	0.3 (0.1; 0.4)	0.00
**Area** (Urban)	28.0 (27.0; 29.0)	27.3 (26.1; 28.4)	30.0 (28.1; 31.9)		23.2 (21.5; 24.9)	30.0 (28.8; 31.2)	
(Rural)	72.0 (71.0; 73.0)	72.7 (71.6; 73.9)	70.0 (68.1; 71.9)	0.01	76.8 (75.1; 78.5)	70.0 (68.8; 71.2)	0.00
**Education** (Primary)	11.6 (10.9; 12.3)	10.8 (10.0; 11.5)	13.8 (12.3; 15.2)		12.9 (11.5; 14.2)	11.0 (10.2; 11.8)	
(Secondary)	72.8 (71.8; 73.7)	73.8 (72.7; 74.9)	69.9 (68.0; 71.8)		74.3 (72.5; 76.0)	72.1 (71.0; 73.3)	
(Higher)	13.8 (13.1; 14.6)	13.7 (12.8; 14.6)	14.3 (12.8; 15.7)	0.00	10.3 (9.1; 11.6)	15.3 (14.4; 16.2)	0.00
**Wealth index quintile** (Poorest)	32.7 (31.7; 33.8)	32.6 (31.4; 33.8)	33.1 (31.1; 35.0)		40.4 (38.4; 42.3)	29.6 (28.4; 30.8)	
(Second)	18.9 (18.0; 19.7)	18.5 (17.5; 19.5)	19.9 (18.2; 21.6)		17.6 (16.0; 19.1)	19.4 (18.4; 20.5)	
(Middle)	16.8 (16.0; 17.6)	17.3 (16.3; 18.3)	15.5 (13.9; 17.0)		15.7 (14.3; 17.2)	17.3 (16.3; 18.2)	
(Fourth)	16.2 (15.4; 17.1)	16.4 (15.4; 17.3)	16.0 (14.4; 17.5)		13.9 (12.6; 15.3)	17.2 (16.2; 18.2)	
(Richest)	15.3 (14.5; 16.1)	15.2 (14.3; 16.2)	15.6 (14.1; 17.1)	0.27	12.4 (11.1; 13.7)	16.6 (15.6; 17.5)	0.00

*N = 8,080 (95% CI)*.

**Figure 1:**
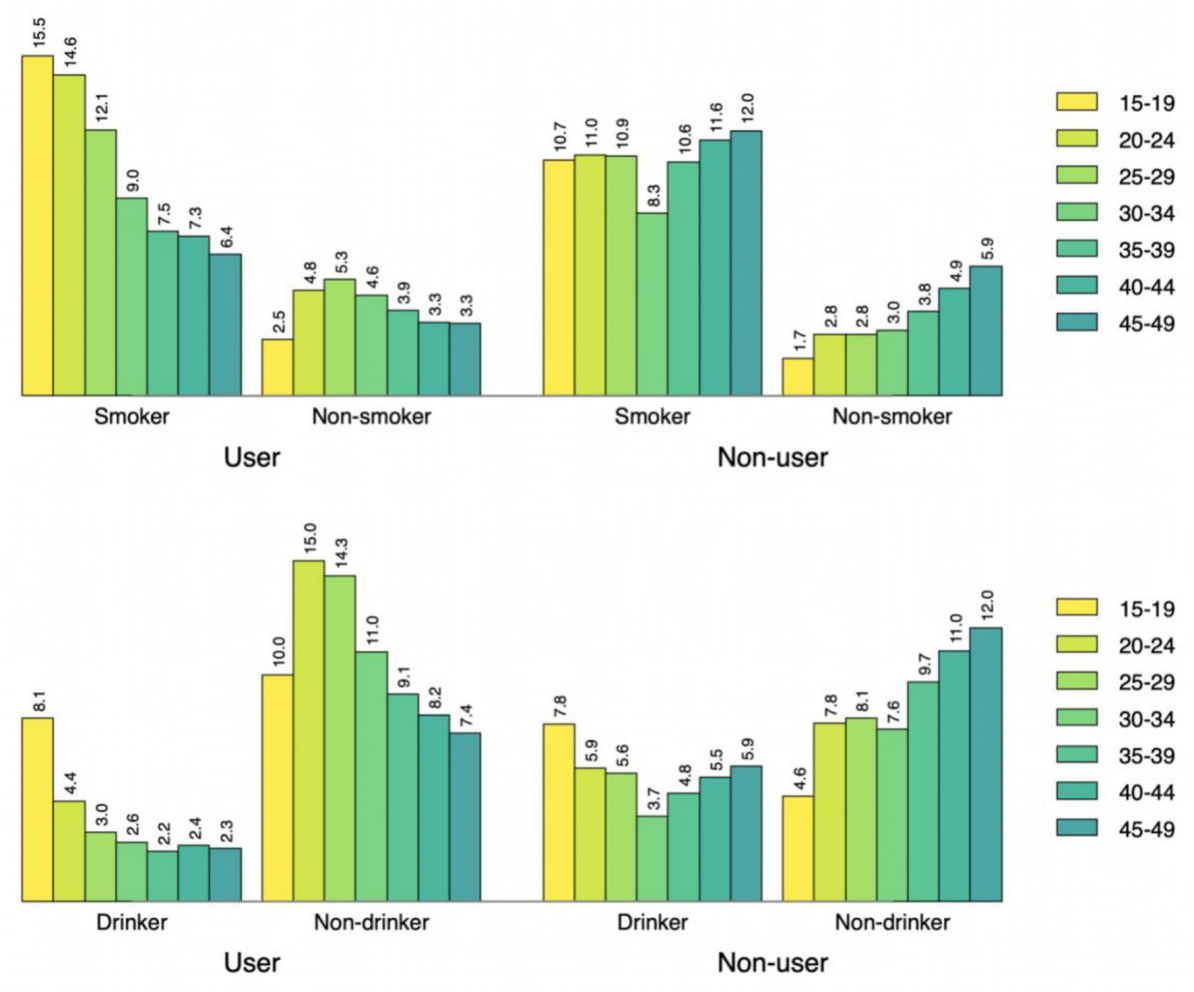
Smoking status across the age groups by internet use status.

**Figure 2:**
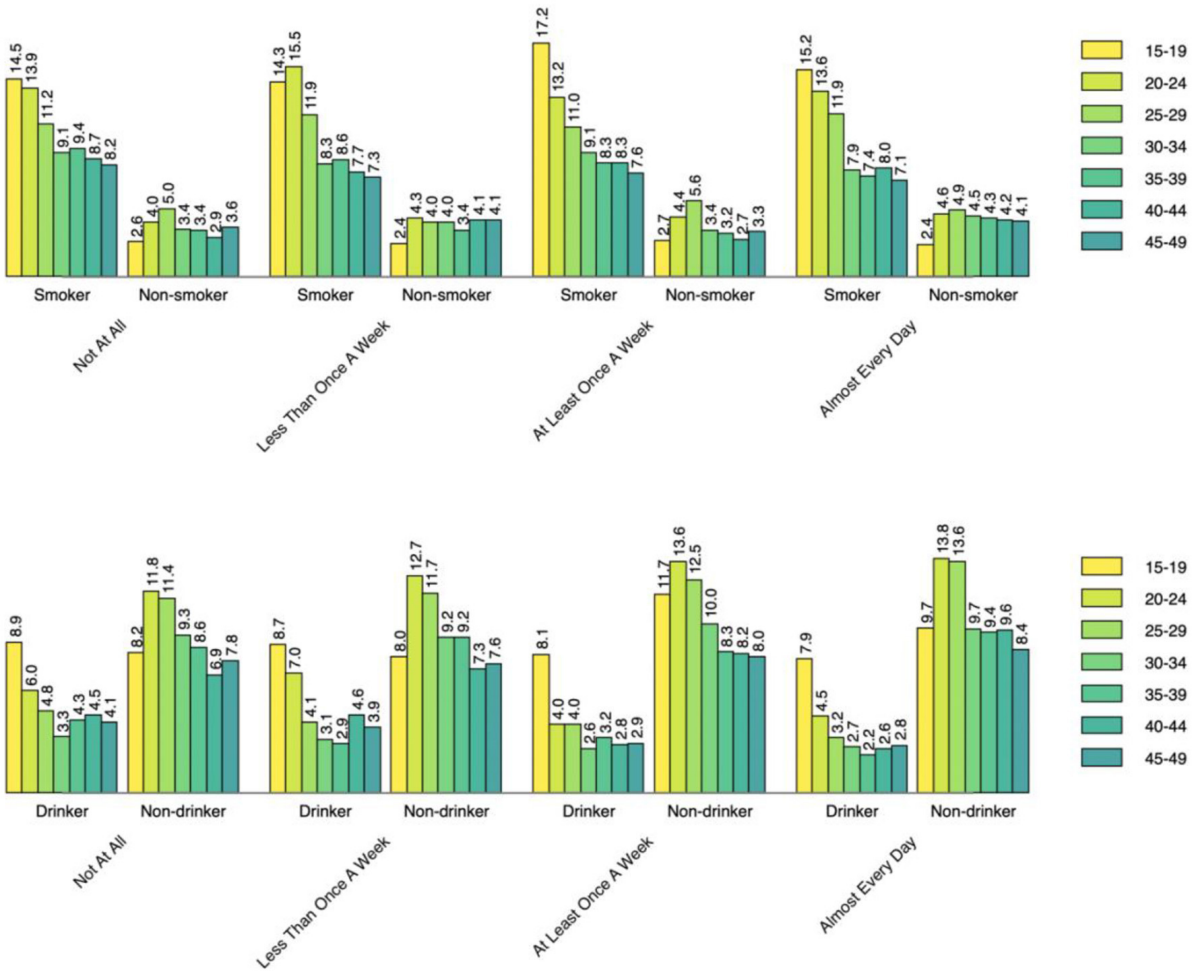
Smoking status across the age groups by TV watching status.

**Figure 3:**
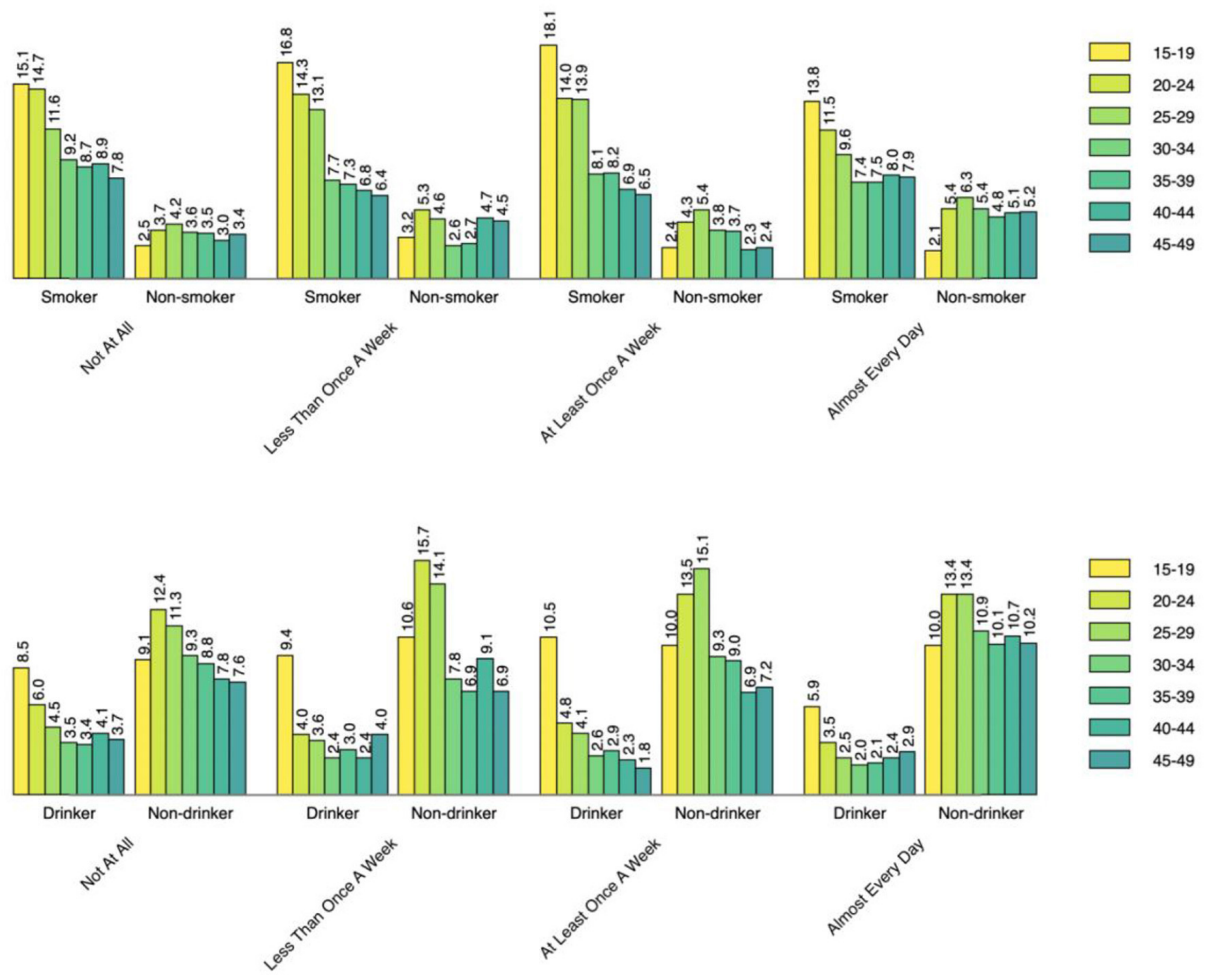
Smoking status across the age groups by radio use status.

Figures 1–3 show the percentages of tobacco and alcohol consumption across the age groups stratified by three types electronic media use status. Among the participants who were internet users, the percentage of smoking and drinking increased gradually with decreasing age groups. The pattern was about the same among TV and radio users as well, however, the group differences (e.g., smoker vs. non-smoker) were less prominent for different frequencies of TV watching and listening to radio than it was for internet use.

Results of multivariable regression analyses show that internet users had significantly higher odds of ever consuming tobacco (Table 2) and alcohol (Table 3). However, the association between internet use and smoking and drinking was not uniform across the sociodemographic variables. For smoking, the positive association with internet use was observed for women only (OR = 1.32, 95% CI = 1.09, 1.59), whereas, for alcohol consumption the association with internet use was significantly positive among both men (1.82, 95% CI = 1.22, 2.70) and women (OR = 1.82, 95% CI = 1.58, 2.09). Men who watched TV at least once a week had lower odds of smoking (OR = 0.65, 95% CI = 0.45, 0.93), and women who listened to radio at least once a week also had lower odds of smoking (OR = 0.63, 95% CI = 0.47, 0.83).

**Table 2:** Association between electronic media use and tobacco smoking among adult men and women in Guyana (2019–20).

	**Full sample**	**Men**	**Women**
**Internet user (No)**	Ref	Ref	Ref
Yes	1.16^*^ [1.01, 1.35]	0.96 [0.75, 1.24]	1.32^**^ [1.09, 1.59]
**TV user (Not At All)**	Ref	Ref	Ref
Less than once a week	0.92 [0.71, 1.20]	0.90 [0.57, 1.41]	0.87 [0.62, 1.23]
At least once a week	0.89 [0.72, 1.09]	0.65* [0.45, 0.93]	1.01 [0.78, 1.31]
Almost every day	1.01 [0.85, 1.21]	0.77 [0.56, 1.06]	1.06 [0.85, 1.33]
**Listens to radio (Not At All)**	Ref	Ref	Ref
Less than once a week	1.05 [0.83, 1.31]	1.22 [0.83, 1.79]	0.92 [0.68, 1.24]
At least once a week	0.75^**^ [0.61, 0.92]	0.92 [0.67, 1.28]	0.63^**^ [0.47, 0.83]
Almost every day	1.56 [0.91, 2.65]	1.26 [0.95, 1.68]	1.16 [0.95, 1.41]

*N.B. Odds ratios with 95% confidence intervals in [] brackets. Models adjusted for all sociodemographic variables*.

*Level of significance*:

* *p < 0.05*,

** *p < 0.01*.

**Table 3:** Association between electronic media use and alcohol drinking among adult men and women in Guyana (2019–20).

	**Full sample**	**Men**	**Women**
**Internet user (No)**	Ref	Ref	Ref
Yes	1.80^***^ [1.58, 2.06]	1.82^***^ [1.58, 2.09]	1.84^**^ [1.22, 2.70]
**TV user (Not At All)**	Ref	Ref	Ref
Less than once a week	0.84 [0.66, 1.06]	0.76 [0.34, 1.68]	0.88 [0.68, 1.13]
At least once a week	1.21^*^ [1.01, 1.46]	1.13 [0.65, 1.99]	1.22^*^ [1.00, 1.49]
Almost every day	1.15 [0.98, 1.36]	0.81 [0.50, 1.32]	1.16 [0.98, 1.38]
**Listens to radio (Not At All)**	Ref	Ref	Ref
Less than once a week	1.10 [0.89, 1.36]	1.06 [0.57, 1.97]	1.06 [0.84, 1.32]
At least once a week	0.97 [0.81, 1.16]	0.77 [0.47, 1.27]	0.99 [0.82, 1.20]
Almost every day	1.15 [0.98, 1.35]	1.14 [0.71, 1.83]	1.11 [0.94, 1.32]

*N.B. Odds ratios with 95% confidence intervals in [] brackets. Models adjusted for all sociodemographic variables. Level of significance*:

* *p < 0.05*,

** *p < 0.01*,

*** *p < 0.001*.

As shown in Table 3, internet use was positively associated with alcohol drinking both among men (OR = 1.82, 95% CI = 1.58, 2.09) and women (OR = 1.84, 95% CI = 1.22, 2.70). Listening to radio didn't show any association with alcohol drinking; however, men who watched TV at least once a week had (OR = 1.22, 95% CI = 1.00, 1.49) times higher odds of ever trying alcohol drinking.

**Figure 4:**
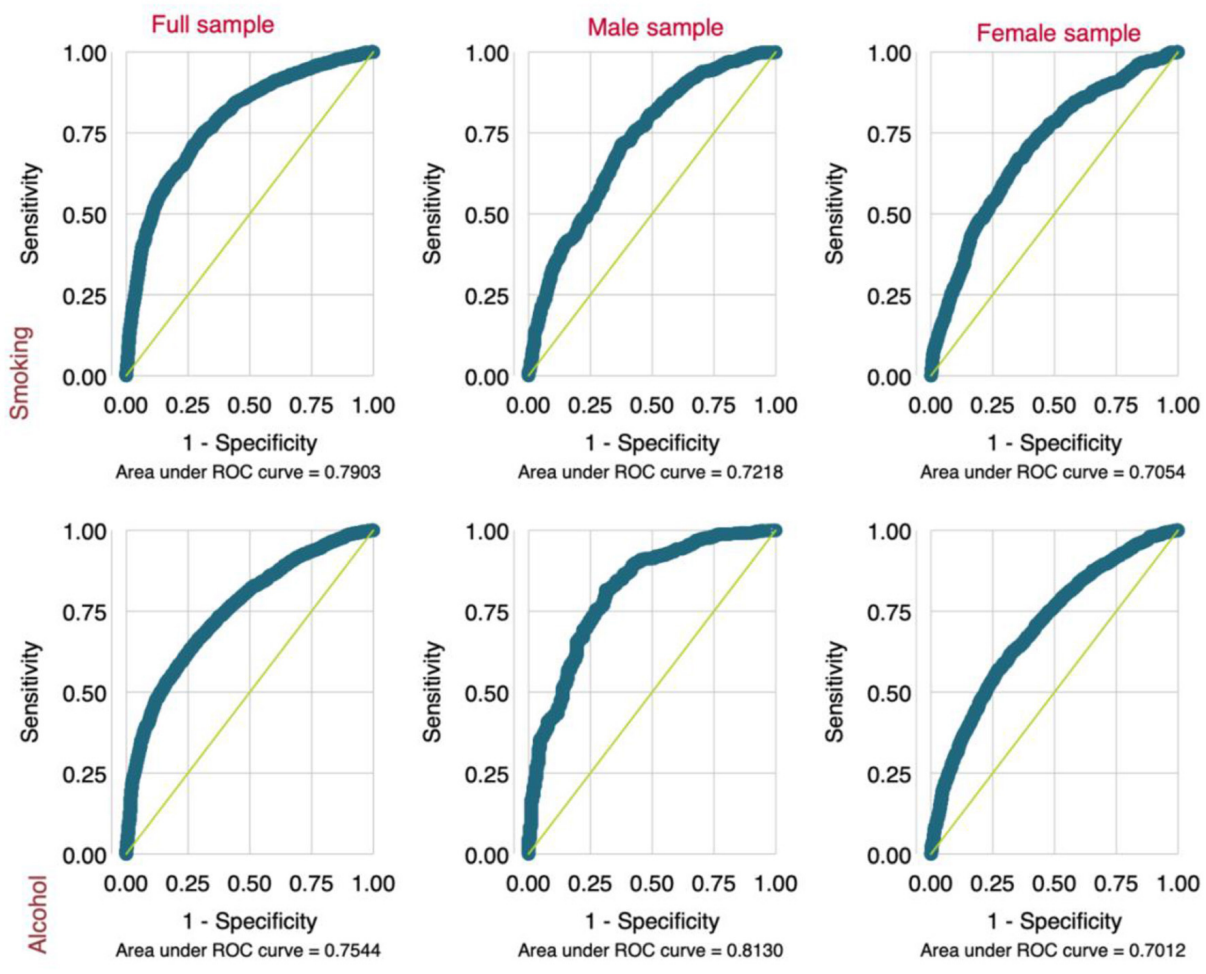
ROC curve values of the regression models.

Regarding the predictive capacity of the regression analyses, all the models had accuracy ranging from 70 to 81% (Figure 4), indicating that the models performed fairly well in predicting the outcome measures.

Table 4 indicates a strongly positive association between tobacco and alcohol consumption among both men and women, with the effects sizes being noticeably higher among women. The associations were significant even after controlling for the sociodemographic factors. In the adjusted model, men and women who drink alcohol had, respectively, 4.6 (OR = 4.64, 95% CI = 3.16, 6.82) and 10.6 times (OR = 10.62, 95% CI = 7.83, 14.40) higher odds of reporting smoking.

**Table 4:** Association between ever trying alcohol drinking and tobacco smoking.

	**Full sample**	**Men**	**Women**
**Unadjusted models**			
**Ever tried alcohol (No)**
Yes	13.34^***^ [10.83, 16.45]	6.80^***^ [4.88, 9.47]	12.85^***^ [9.66, 17.09]
**Models adjusted for all covariates**			
**Ever tried alcohol (No)**
Yes	8.30^***^ [6.58, 10.46]	4.64^***^ [3.16, 6.82]	10.62^***^ [7.83, 14.40]

*N.B. Odds ratios with 95% confidence intervals in [] brackets. Level of significance*:

^*^p < 0.051,

^**^p < 0.01,

*** *p < 0.001*.

## Discussion

Based on data from the Guyana Multiple Indicator Cluster Survey, this study aimed to the assess the relationship between electronic media use and tobacco and alcohol consumption behavior among adult men and women in Guyana. Our findings indicate that more than a quarter of the participants (27.1%) ever smoked tobacco while more than three-fifths ever drank alcohol. The percentage of tobacco consumption is higher compared with the that in the USA (20.8%) in 2019 (31), but that of lifetime alcohol consumption is lower (32). After stratifying the descriptive analysis by age and internet use status, those in the younger age groups were found to have higher percentage of smoking and alcohol drinking. Interestingly, the percentage of both smoking and drinking was lowest among oldest age group of 45–49 years, which might be because the younger population are more likely to use internet than and acquire new social or health-related behavior from peers. Similar to internet use, the percentage of smoking and drinking was higher among the youngest TV and radio users. Participants who watch TV almost every day had the highest percentage of reporting tobacco and alcohol use. In contrast, those who never listen to radio had the lowest percentage of reporting tobacco and alcohol use. The possible reason might be that TV watching involves a more sedentary or leisurely activity that encourages behavior regardless of the content (33). Previous research have reported increased food and beverage intake during TV watching (34), however that of smoking and drinking is yet to be explored.

Results of regression analysis showed a strong link between exposure to electronic media and tobacco and alcohol consumption among both men and women. Internet use was positively associated with smoking for women only and association was not significant for men. For alcohol consumption, there was a strong positive association with internet use was significantly positive among both men and women. In contrast to internet use, the associations between TV watching and listening to radio with tobacco and alcohol was did not show any noticeable pattern. For instance, watching TV at least once a week showed a protective effect against smoking among men only, and the associations were not significant for other frequencies of TV watching. Similarly, listening to radio at least once a week showed a protective effect against smoking among women but not among men. Unlike for smoking, TV watching at least once a week showed a strong positive association with drinking among women. We found no significant association between listening to radio and alcohol drinking either in the full or in the sex-stratified sample. In short, among all the types of electronic media, only internet use showed a positive association with smoking among men, and both smoking and drinking among women.

The findings on these associations should however be interpreted with caution since the likely explanations or mechanisms that underlie them are not clear from the current data. According to the existing studies, one possible mechanism could be exposure to tobacco and alcohol related posts from peers, TV programs, or commercial advertisers (35–37). Apart from the association between electronic and tobacco and alcohol consumption, our study also aligns with the existing literature regarding the influence of alcohol and on smoking (38, 39). Even after controlling for the potentially confounding sociodemographic and media use factors, we observed remarkably high odds of smoking among both men and women who drank alcohol. Alcohol drinkers are more likely to smoke as growing higher tolerance for tobacco may induce higher tolerance for drinking, and vice-versa (40). Thus, it is suggestible that anti-tobacco campaigns emphasize the prevention of alcohol consumption at the same time. Globally, NCDs constitute a contributor to overall morbidity and mortality to which Guyana is no exception. Tobacco and alcohol consumption are two leading risk factors of NCDs, and therefore their consumption should be curbed through evidence-based interventions such as minimizing the exposure of TV programs and advertisements that tend to glamourize these unhealthy behaviors.

Socioeconomic determinants of tobacco smoking and alcohol drinking are well-documented, but literature on the influence of electronic media on these behaviors is still mixed. Our analyses suggest that exposure to electronic media is strongly associated with ever trying alcohol and tobacco use among Guyanese men and women. We conducted the regression analyses in two steps, first by adding only the variables of interest, and finally by adjusting for all covariates to examine whether the addition of the socioeconomic factors make any difference in the associations. The results suggest that adjusting the socioeconomic factors reduced the effect sizes by a small margin, implying a potential role of these factors in smoking and drinking behavior in this population. In addition, stratifying the analyses for male and female samples allowed us to understand the gender differences in the association. Gender is a key demographic factor in lifestyle medicine literature and previous studies have documented gender differences in almost all behavioral factors including smoking, drinking and electronic media use (41–43). Our findings add to the literature that use of electronic media may have a stronger influence on ever trying smoking and alcohol drinking among women than among men in Guyana that can inform smoking and alcohol prevention programs in the country.

This study has several important limitations to report. Firstly, the data were secondary and thus we have no control over the selection and measurement of the study variables. It is possible that the associations are due to residual confounding of factors that the analysis was not controlled for. Although the analysis included the key sociodemographic and media use related variables, several other influential factors such peer and family environment were not accounted for which were found to be important predictors of tobacco and alcohol use (44, 45). Secondly, there was no data on the amount of tobacco and alcohol consumption and hence we were unable to examine whether there is any dose-response relationship between electronic media use and alcohol and tobacco consumption in the sample population. There was also no data on the actual content seen or heard on the three types electronic media that we included in the study, and therefore we were unable to ascertain whether the participants were exposed to peers' substance-related posts or company advertisements. Thirdly, data were self-reported and hence remain subject to reporting biases. Lastly the survey was cross sectional in nature, and therefore the associations preclude making any causal relationship. Regardless of these limitations, the present study makes an important contribution to the literature. This is the first study on tobacco and alcohol use in a nationally-representative population in Guyana.

## Conclusion

Our analysis of the Multiple Indicator Cluster Survey data revealed a high prevalence of lifetime tobacco and alcohol consumption among Guyanese men and women, with the prevalence of alcohol consumption being remarkably higher than that of tobacco. Overall, more than a quarter of the participants reported ever smoking tobacco and more than three-fifths ever drinking alcohol. Exposure to electronic media, especially that of internet was found to be significantly associated with both tobacco and alcohol consumption among both men and women. We also found a strong interrelation between alcohol and tobacco smoking, meaning that tobacco control policies should pay particular attention to alcohol users. Further studies on electronic media content and context are necessary to better understand the mechanisms by which their exposure may trigger tobacco and alcohol consumption in this population.

## Data Availability Statement

Publicly available datasets were analyzed in this study. This data can be found here: https://mics.unicef.org/surveys.

## Author Contributions

LS: conceptualization. LS, TZ, BG, and LJ: data collection and analysis. LS, TZ, YC, and LJ: writing and reviewing. All authors contributed to the article and approved the submitted version.

## Conflict of Interest

The authors declare that the research was conducted in the absence of any commercial or financial relationships that could be construed as a potential conflict of interest.

## Publisher's Note

All claims expressed in this article are solely those of the authors and do not necessarily represent those of their affiliated organizations, or those of the publisher, the editors and the reviewers. Any product that may be evaluated in this article, or claim that may be made by its manufacturer, is not guaranteed or endorsed by the publisher.
